# Characteristics of adolescents referred for bariatric surgery in Abu Dhabi, United Arab Emirates

**DOI:** 10.3389/fped.2024.1297251

**Published:** 2024-03-08

**Authors:** Reem Hassan Beck, Imrana Afrooz, Muhammad Suhail Masalawala, Rama Watad, Talat Al Shaban, Asma Deeb

**Affiliations:** ^1^ Clinical Trial Unit, Sheikh Shakhbout Medical City, Abu Dhabi, United Arab Emirates; ^2^ Paediatric Endocrine Division, Sheikh Shakhbout Medical City, Abu Dhabi, United Arab Emirates; ^3^ Department of Surgery, Sheikh Shakhbout Medical City, Abu Dhabi, United Arab Emirates

**Keywords:** adolescent, body mass index, metabolic and bariatric surgery, obesity, United Arab Emirates

## Abstract

About a third of children and adolescents are overweight or obese in the United Arab Emirates, and referrals for metabolic and bariatric surgery (MBS) are now common. Despite excellent evidence that MBS should be considered in adolescents with severe obesity, it remains a management approach of last resort in many cases. Baseline, real-world data on adolescent patients living with obesity referred for surgery, their characteristics, and how these relate to current and future referral policy are important to ensure best practice. Here we examined the demographic, anthropometric, and clinical characteristics of adolescents referred for MBS over a three-year period to Sheikh Shakhbout Medical City (SSMC), Abu Dhabi, UAE. Ninety-two adolescents living with obesity were recruited: 54.3% were female, the average age was 16.3 ± 2.4 years, and 88.0% of patients had a first-degree relative with a history of obesity and 62% a family history of bariatric surgery. The average BMI was 47.7 ± 10.5, and the average percentage of the 95th percentile BMI was 169.5 ± 38.8%. Complications of obesity (hypertension, type 2 diabetes and prediabetes, dyslipidemia, and liver function abnormalities) were common. Our analysis highlights that there exists a mismatch between the profiles of patients referred for MBS, local guidelines, and international best practice in decision-making for referral to MBS services. While many adolescents in the UAE seem to enjoy family support and experience in the surgical management of obesity, local guidelines need updating to reflect changes in the definitions of obesity, thresholds for referral, and to remove unnecessary developmental stage barriers to increase the window for personalized surgical management.

## 1 Introduction

Obesity is a major public health challenge across the world, not least in the United Arab Emirates (UAE), where about a third of children and adolescents are classified as overweight or obese (1). Obesity is multifactorial and driven by genetics (2) and multiple socioeconomic, geographic, and environmental factors, especially individual and parental behaviors (3). The combined effects of these risk factors have driven a steady increase in the prevalence of childhood obesity globally and in the UAE over the last twenty years (3), especially in school-aged children in the 11–14-year age group (4). The recent COVID-19 pandemic only served to further exacerbate the problem in the UAE, with distance learning during lockdown increasing unhealthy food consumption and snacking (5). Obesity is a lifelong determinant of chronic ill-health and disease including metabolic disorders, cardiovascular disease, and cancer (6, 7), mandating robust responses to prevent and treat the condition. Given the well-established associations between obesity and these severe complications (8) and that these diseases tend to have a more aggressive clinical course the earlier they develop in life (9), the long-term health and wellbeing of children and adolescents living with obesity relies on appropriate, optimal, and personalized management of the disease in childhood to prevent a lifetime of physical and psychological ill-health.

Despite encouraging recent data showing decreases in BMI with glucagon-like peptide-1 receptor agonists in adolescents with obesity (10), non-surgical treatments for obesity such as diet, exercise, and behavioral modifications have generally been disappointing, with only modest outcome benefits in both adults and children (11–13). In adults, it is now accepted that metabolic and bariatric surgery (MBS) is the most effective therapy for individuals with a BMI ≥40 kg/m^2^ or BMI ≥35 kg/m^2^ with obesity-related co-morbidities (14) and even for individuals with a BMI between 30 and 34 kg/m^2^ with uncontrolled diabetes (15). There is also a growing body of evidence that MBS is efficacious in the adolescent age group [10–19 as defined by the WHO (16)]. Prospective longitudinal studies have shown that MBS results in significant and persistent reductions in body weight, correction of metabolic abnormalities, and increased quality of life with excellent patient satisfaction and safety profiles (17–20). This high-quality evidence has prompted support and evidence-based guidelines from the American Society for Metabolic and Bariatric Surgery (ASMBS) on the indications for MBS in the adolescent population (16), which largely mirror those for adults (21).

Despite this evidence, there remain barriers to the adoption of MBS in the routine care of adolescents with obesity, meaning that it remains a management approach of last resort in many cases. These barriers include persistent stigma associated with obesity, including among healthcare professionals; a lack of education about the positive benefits of surgery in the treatment of childhood obesity; and a lack of data on the prevalence and clinical characteristics of adolescents living with obesity and suitable for MBS. Furthermore, local guidelines on the management of adolescents living with obesity—including those in the UAE (22, 23)—may not accurately reflect best practice or expert consensus as reflected in the evidence-based guidelines. For instance, current UAE guidelines still mandate epiphyseal closure as a requirement for MBS (22, 23), despite an absence of evidence that puberty status is adversely affected by MBS. Therefore, capitalizing on the high prevalence of childhood obesity in our pediatric practice, we examine the demographic, anthropometric, and clinical characteristics of adolescents referred for MBS over a three-year period to provide new, real-world data describing the clinical need for MBS in this growing population of high-risk patients.

## 2 Methods

This study is reported according to the STROBE guidelines for observational studies (24). This was a prospective observational, cohort study of adolescents living with obesity attending pediatric endocrine clinics as part of their multidisciplinary team management as candidates for bariatric surgery between January 2020 and July 2023 at Sheikh Shakhbout Medical City (SSMC), Abu Dhabi, UAE. Patients were aged between 12 and 19 years according to the WHO definition of adolescence (16). The Institutional Review Board of the SSMC approved the study protocol (protocol number SSMCREC-322), and all children or their parents/guardians provided written consent or assent for study participation.

Patients were selected according to rigorous, structured local practice (22, 23). For eligibility for bariatric surgery, they must have failed intensive lifestyle interventions for a minimum of six months under comprehensive, structured supervision by the multidisciplinary team and they had to meet the following criteria: (i) be severely obese (defined by the World Health Organization as a body mass index >40); (ii) have comorbidities related to obesity that might be remedied with durable weight loss; and (iii) show skeletal and developmental maturity. For patients prescribed liraglutide, this was only administered short-term (–12 weeks), as the drug is not covered by insurance in the UAE, precluding long-term treatment or achieving the higher effective dose.

Electronic medical records (EMR) were reviewed to obtain: (i) demographic data (sex, age at referral); (ii) clinical history [history of anti-obesity medication use, family history of obesity and history of bariatric surgery in a first degree relative (parent or sibling)]; (iii) anthropometric measures of weight (to the nearest 0.1 kg), height (to the nearest 0.1 cm), and BMI (calculated as kilograms divided by meters squared), with weight and BMI z-scores and centiles obtained through formulae embedded the EMR based on CDC growth charts; (iv) systolic and diastolic blood pressure recorded and interpreted as normal, elevated, stage 1, or stage 2 hypertension as per (25); (v) fasting lipid profile (total cholesterol, low density lipoprotein (LDL), high density lipoprotein (HDL), and triglycerides interpreted as per guidelines (26); (vi) HbA1c classified as normal if below 5.7%, pre-diabetes between 5.7% and 6.4%, and type 2 diabetes if over 6.4% (27); and (vii) presence or absence of obstructive sleep apnea (OSA). Due to inaccuracies in CDC z-scores and percentiles at very high BMIs in children (28, 29), obesity was also defined according to percentage BMI over the 95th percentile (class I, II and III obesity).

Data were analyzed in SPSS v.28 (IBM Statistics, Armonk, NY) and are presented as means (standard deviations; SD) or counts (%) for continuous or categorical data, respectively.

## 3 Results

Ninety-two adolescents living with obesity were recruited to the study, and their clinical, demographic, and anthropometric characteristics are presented in Table 1. Just over half (54.3%) were female, and the average age of the study population was 16.3 ± 2.4 years. The majority of patients had a first-degree relative with a history of obesity (88.0%), and nearly two-thirds (62%) had a family history of bariatric surgery: in six patients (6.5%), both parents had received surgery and in eleven patients (11.9%), two or more siblings had received bariatric surgery. Nine patients had previously been prescribed anti-obesity medication (liraglutide, 9.8%). However, as the drug is not covered by insurance in the UAE, liraglutide was only prescribed short-term in these patients, precluding long-term treatment or achieving the higher effective dose.

**Table 1:** Demographic and weight characteristics of adolescents living with obesity referred for bariatric surgery in the United Arab Emirates (*n *= 92).

Variable		Mean (SD)/*N* (%)
Age at referral (years)		16.3 (2.4)
Gender	Male	42 (45.7)
	Female	50 (54.3)
History of anti-obesity medication	Nil	83 (90.2)
	Liraglutide	9 (9.8)
Family history of obesity	Yes	81 (88.0)
	No	11 (12.0)
Family history of bariatric surgery	Yes	57 (62.0)
	No	35 (38.0)
Weight at presentation (kg)		126.3 (32.1)
BMI		47.7 (10.5)
% 95th percentile BMI		169.5 (38.8)
BMI z-score		2.72 (0.33)
BMI centile		99.5 (0.44)

Nearly all patients had class 2 (*n* = 21, 22.8%; defined as a BMI ≥120% to <140% of the 95th percentile or BMI ≥35–<40 kg/m^2^) or class 3 (*n* = 71, 77.2%; defined as a BMI ≥140% of the 95th percentile or BMI ≥40 kg/m^2^) obesity (3, 29). The average BMI was 47.7 ± 10.5, and the average percentage of the 95th percentile BMI was 169.5 ± 38.8%.

Complications of obesity were common in the study population (Table 2). Half had OSA, and nearly half (43.5%) had established hypertension, ten (10.9%) with stage 2 disease. Although only three patients (3.3%) had established type 2 diabetes, nearly two-fifths had prediabetes (HbA1c 5.8%–6.5%). Dyslipidemia was common: two-fifths to a third of patients had borderline abnormal cholesterol, LDL, HDL, and triglyceride levels, while 5.4%, 3.3%, and 21.7% had abnormally high cholesterol, LDL, and triglyceride levels, respectively. Over a quarter of patients (28.3%) had a high ALT level indicative of steatohepatitis.

**Table 2:** Metabolic characteristics of the study cohort and frequency of complications (*n* = 92).

Variable		Mean (SD)/*N* (%)
Obstructive sleep apnea	Yes	46 (50)
	No	46 (50)
Hypertension	Nil	39 (42.4)
	Elevated	13 (14.1)
	Stage 1	30 (32.6)
	Stage 2	10 (10.9)
Type 2 diabetes	Nil	53 (57.6)
	Prediabetic	36 (39.1)
	Type 2 diabetes	3 (3.3)
HbA1c (5)		5.6 (0.43)
Total cholesterol (mmol/L)	Mean (SD)	4.0 (0.73)
	Normal	64 (69.6)
	Borderline	23 (25.0)
	High	5 (5.4)
LDL (mmol/L)	Mean (SD)	2.32 (0.58)
	Normal	71 (77.2)
	Borderline	18 (19.6)
	High	3 (3.3)
HDL (mmol/L)	Mean (SD)	1.23 (0.28)
	Normal	49 (53.3)
	Borderline	28 (30.4)
	Low	15 (16.3)
Triglycerides (mmol/L)	Mean (SD)	1.18 (0.54)
	Normal	53 (57.6)
	Borderline	19 (20.7)
	High	20 (21.7)
ALT	Normal	66 (71.7)
	High	26 (28.3)
Number of comorbidities	None	6 (6.5)
	1	26 (28.3)
	2	15 (16.3)
	3	19 (20.7)
	4	12 (13.0)
	5	7 (7.6)
	6	4 (4.3)
	7	3 (3.3)

Finally, we enumerated the number of complications (OSA, hypertension, borderline/high cholesterol, LDL, and triglyceride, low HDL, and abnormal ALT) in the study population, and nearly half of patients (48.9%) had three or more complications. Even when defined according to these weakly stringent criteria to include borderline lipid and metabolic abnormalities, three patients with class 3 obesity (4.2%) had no biochemical or clinical evidence of obesity-related complications.

## 4 Discussion

Baseline, real-world data on adolescents with obesity referred for surgery, their characteristics, and how these relate to current and future referral policy are important to ensure best practice. Here we describe real-world data on the characteristics of adolescents referred for MBS in a tertiary center in the UAE. Over a three-year period, nearly a hundred adolescents were referred for the procedure, highlighting the frequency and severity of the problem. Children referred for MBS lived with severe class 2 or 3 obesity, had frequent family histories of obesity and bariatric surgery, and experienced high levels of comorbidities including hypertension, dyslipidemia, OSA, and diabetes. However, not all children deemed clinically suitable for MBS in this cohort had a BMI >40, comorbidities, or had reached skeletal maturity as mandated in current UAE Department of Health Weight Management Program and Surgical Management guidelines (23). Furthermore, some children with class 3 obesity showed no evidence of obesity-related comorbidities, highlighting that physiological and biochemical measures of comorbidities are imperfect criteria for patient selection. Overall, our data highlight enthusiasm for MBS but also a need to re-examine local referral guidelines considering the growing population of patients in need and updates to international evidence-based guidelines. Doing so would ensure that the window to individualize surgery in adolescents is maximized.

Local management of adolescents living with obesity in the UAE is through rigorous, structured practice (22, 23). According to current guidelines, adolescents with obesity (defined as a BMI >2 SD above the WHO Growth Reference median, equivalent to the 97th percentile) are eligible for family-centered, multidisciplinary, insurance-funded care involving specialist physicians, dieticians, psychologists, and other relevant health professionals as required. For a young patient to be eligible for bariatric surgery, intensive lifestyle interventions must be attempted for a minimum of six months under comprehensive, structured supervision the multidisciplinary team. Furthermore, current DoH standards for the surgical management of obesity state that adolescents (<18 years) must meet all of the following major criteria in addition to six months of intensive lifestyle interventions: (i) be severely obese (defined by the World Health Organization as a body mass index >40); (ii) have comorbidities related to obesity that might be remedied with durable weight loss; and (iii) show skeletal and developmental maturity. Therefore, while UAE guidelines for adults mirror current international guidance of BMI ≥40 kg/m^2^ without comorbidities or BMI ≥35 kg/m^2^ with obesity-related co-morbidities (14) and even for individuals with a BMI between 30 and 34 kg/m^2^ with uncontrolled diabetes (15), the acceptance criteria for adolescents currently do not reflect current evidence-based best practice in four main areas: (i) definition of adolescence; (ii) definitions of childhood obesity; (iii) requirements for comorbidities in class 3 disease; and (iv) the requirement for skeletal and developmental maturity. These discrepancies with well-accepted ASMBS consensus guidelines risk reducing the number of young people eligible for MBS who might otherwise benefit, thereby placing adolescents living with obesity at a significant disadvantage and increase the risk of persistent obesity and end-organ damage from chronic comorbidities.

First, the ASMBS guidelines define adolescence according to WHO criteria, i.e., people between 10 and 19 years of age (16), compared to an upper limit of 17 in current UAE guidelines.

Second, recognizing that CDC z-scores and percentiles are inaccurate in children and adolescents with very high BMIs (28, 29), the CDC, American Academy of Pediatrics (AAP), and the American Heart Association (AHA) now consistently define obesity categories in adolescents using percentage of the 95th percentile cutoffs, i.e., obesity class I (≥95th percentile to <120% of the 95th percentile); obesity class II (≥120% to <140% of the 95th percentile) or a BMI ≥35–≤39 kg/m^2^, whichever is lower; or obesity class III (≥140% of the 95th percentile) or BMI ≥40 kg/m^2^, whichever is lower (3, 29). In this way, criteria for MBS now mirror the recommendations for adults [BMI ≥40 kg/m^2^ without comorbidities or BMI ≥35 kg/m^2^ with obesity-related co-morbidities (14)], albeit with a slightly extended range of comorbidities specific for adolescents (hyperlipidemia, hypertension, type 2 diabetes, insulin resistance, reduced health-related quality of life, gastroesophageal reflux disease, OSA, non-alcoholic steatohepatitis, orthopedic disease, idiopathic intracranial hypertension).

Third, requiring adolescents with a BMI >40 to have a co-morbidity (as in current local and older guidelines) places young people at unnecessary risk of future [and more severe (9)] health risks. Our data show that not all patients with class 3 obesity have overt comorbidities, but this does not necessarily correspond to a reduced risk of future complications, rapid deterioration of less severe complications, nor that MBS would be ineffective. The reasons why some patients with class 3 obesity develop complications and others do not require further clarification, not least because the development of complications may at least in part be genetically determined [such as through specific polymorphisms, e.g., in the fat mass and obesity-associated gene, *FTO* (30)] and therefore useful for personalized management. It might also in part explain why the data evidencing the health risks from obesity-related comorbidities are unequivocal. Severe obesity is associated with the development of several cardiovascular risk factors such as type 2 diabetes and dyslipidemia, subsequent cardiovascular disease, and premature mortality through cardiovascular events in early adulthood (31, 32). Importantly, children who become non-obese by adulthood no longer carry the same risks of adverse cardiovascular outcomes (32), including when weight loss is attainted through MBS (33). When type 2 diabetes develops in adolescents, β-cell function declines more rapidly than in adulthood (34), end-organ injury occurs earlier (35), and both primary disease and its complications tend to be medical therapy-resistant (36). Most importantly, MBS has been shown to promote very high rates of remission of type 2 diabetes, even more so in adolescents than in adults (17, 37). Similar impressive and persistent resolutions of comorbidities after MBS in adolescents have been observed for OSA (38, 39) and quality of life (16, 17).

Fourth, there is no evidence that withholding MBS from children and adolescents who have yet to undergo or complete puberty is of benefit. On the contrary, one case-control study comparing children undergoing MBS with age- and sex-matched controls receiving non-surgical management showed faster growth in the group undergoing surgery (40). A recent analysis comparing younger (13–15 years) and older (16–19 years) adolescents similarly showed similar outcomes in terms of hypertension and dyslipidemia remission with equivalent weight loss and quality of life improvements (41). Although more prospective, longitudinal data are required, given the clear benefit-to-risk profile of MBS, physicians and pediatricians should no longer hesitate to refer younger adolescents for MBS through fear of adverse effects on growth and development (42).

The frequency of comorbidities in our cohort was similar to that seen in other populations (16, 17). However, the demographics of our population differed slightly to those observed in other countries and studies, especially with regard to the age and sex distributions. In our cohort, there was an approximately equal distribution of males and females, compared with many previous studies from the US that have consistently reported a majority of female patients (70% or more) (43–45). The reasons for this difference are unclear, although a higher acceptance (i.e., preference) for MBS has been reported in girls than boys in a Swedish study, perhaps because girls have a higher psychosocial burden from obesity than boys, motivating acceptance of invasive procedures (46). While this might also be true in the UAE, our population was enriched for patients with first-degree relatives who were also obese or who had undergone bariatric surgery. The reasons for the strong family history seen in our population are unclear, and we did not perform genetic testing to investigate for uncommon monogenic forms. However, given the complex and multifactorial nature of obesity (47), the familial predisposition is likely to represent a mixture of inheritance of low- to moderate-risk susceptibility genes [there is good evidence that energy homeostasis and thermogenesis, adipogenesis, leptin-insulin signaling, and hormonal signaling genes contribute to the development of obesity (48, 49)] and exposure to a common “obesogenic environment”, which is prominent in Arab countries (50). Nevertheless, it has been shown that the decision to pursue surgery and subsequent behavior is significantly influenced by family in the Middle East, where there is greater involvement of family in healthcare decision-making (51). These cultural and family influences may influence positive decision-making in children and adolescents living with obesity. Furthermore, compared with previous US studies where the majority (>80%) of adolescents undergoing MBS were 17–19 years of age (43, 52), over half (55/92, 60%) of our cohort were aged <17 years. Far from indicating hesitancy for surgery from younger patients and their families, there seems to be greater acceptance of MBS for younger adolescents in the UAE. Provided that the medical professional can similarly follow suit and reduce barriers to surgery for prepubertal children with obesity, there seems to be a culture of acceptance of MBS in the UAE that could help contribute to improving outcomes for adolescents with severe obesity.

This study has some limitations. First, this was a single-center, observational study, so there may have been selection biases and the results are not generalizable to other populations. However, the aim of this study was to provide insights into the local characteristics of adolescents referred for MBS to educate and inform local referral practice. Second, we did not have data on every comorbidity such as health-related quality of life, gastroesophageal reflux disease, orthopedic disease, and idiopathic intracranial hypertension, so we may not have accounted for every comorbidity in out cohort. Third, we did not assess the psychosocial factors that influenced the decision of patients to pursue MBS, and although we speculate that cultural and family influences may have influenced positive decision-making in children and adolescents living with obesity in the UAE, it would be interesting to quantify the influence of psychosocial factors on patient decision-making. Fourth, we did not perform any genetic analyses, so we could not establish any genetic determinants of obesity and obesity-related complications. Fifth, although the evidence supporting MBS as part of standard-of-care for the management of adolescents living with obesity is now unequivocal, including in the UAE (53), the procedure does have some side-effects including decreased absorption of vitamins and minerals that require proactive clinical management including dietary intervention and supplements to prevent negative clinical outcomes (54), and we did not examine these sequelae here. Finally, only a small proportion of our cohort trialed weight loss medications prior to MBS, and, given the data supporting the efficacy of glucagon-like peptide-1 receptor agonists in adolescents with obesity (55), further work is now required to definitively establish the role for pharmacotherapy in bridging the gap between diet and lifestyle interventions and weight loss surgery.

This study provides new data on the clinical, demographic, and anthropometric profiles of adolescents referred for surgery in the UAE. Our analysis highlights that there is a mismatch between the profiles of patients referred for MBS, local guidelines, and international best practice in decision-making for referral to MBS services. Local guidelines in the UAE for children and adolescents living with obesity have many positive aspects, not least their family-centered, multidisciplinary approach, but they also need updating to reflect changes in the definitions of obesity, thresholds for referral, and unnecessary developmental barriers to increase the window for personalized surgical management. Given that many adolescents in the UAE seem to enjoy family support and experience in the surgical management of obesity, it is imperative that the profession similarly embraces best practice to improve the present and future health of these young people. Further large, prospective, multicenter studies are needed to provide empirical evidence supporting revision of the selection criteria for referral to bariatric surgery and to explore the long-term effects of the procedure on growth, puberty and nutritional status and health in children undergoing bariatric surgery, especially at younger ages.

## Data Availability

The original contributions presented in the study are included in the article/Supplementary Material, further inquiries can be directed to the corresponding author.

## References

[1] Farrag NS Cheskin LJ Farag MK. A systematic review of childhood obesity in the Middle East and North Africa (MENA) region: prevalence and risk factors meta-analysis. Adv Pediatr Res. (2017) 4:8. 10.12715/apr.2017.4.8 29354689 PMC5773115

[2] Littleton SH Berkowitz RI Grant SFA. Genetic determinants of childhood obesity. Mol Diagn Ther. (2020) 24(6):653–63. 10.1007/s40291-020-00496-1 33006084 PMC7680380

[3] Al Sabbah H Assaf EA Al-Jawaldeh A AlSammach AS Madi H Khamis Al Ali N Nutrition situation analysis in the UAE: a review study. Nutrients. (2023) 15(2):363. 10.3390/nu15020363 36678240 PMC9861891

[4] AlBlooshi A Shaban S AlTunaiji M Fares N AlShehhi L AlShehhi H Increasing obesity rates in school children in United Arab Emirates. Obes Sci Pract. (2016) 2(2):196–202. 10.1002/osp4.37 27818779 PMC5074293

[5] Sajwani NH Qawas A Al Ali N Sajwani FH Alrustamani AH Al Maamari S The effect of lockdowns and distant learning on the health-related behaviours of school students in the United Arab Emirates. BMC Prim Care. (2022) 23(1):253. 10.1186/s12875-022-01856-y 36167505 PMC9513288

[6] Daniels SR. Complications of obesity in children and adolescents. Int J Obes (Lond). (2009) 33(Suppl 1):S60–5. 10.1038/ijo.2009.20 19363511

[7] Horesh A Tsur AM Bardugo A Twig G. Adolescent and childhood obesity and excess morbidity and mortality in young adulthood-a systematic review. Curr Obes Rep. (2021) 10(3):301–10. 10.1007/s13679-021-00439-9 33950400

[8] Kelly RK Magnussen CG Sabin MA Cheung M Juonala M. Development of hypertension in overweight adolescents: a review. Adolesc Health Med Ther. (2015) 6:171–87. 10.2147/AHMT.S55837 26543386 PMC4622556

[9] Lascar N Brown J Pattison H Barnett AH Bailey CJ Bellary S. Type 2 diabetes in adolescents and young adults. Lancet Diabetes Endocrinol. (2018) 6(1):69–80. 10.1016/S2213-8587(17)30186-9 28847479

[10] Weghuber D Barrett T Barrientos-Perez M Gies I Hesse D Jeppesen OK Once-weekly semaglutide in adolescents with obesity. N Engl J Med. (2022) 387(24):2245–57. 10.1056/NEJMoa2208601 36322838 PMC9997064

[11] Summerbell CD Ashton V Campbell KJ Edmunds L Kelly S Waters E. Interventions for treating obesity in children. Cochrane Database Syst Rev. (2003) 3:CD001872. 10.1002/14651858.CD001872 12917914

[12] van der Baan-Slootweg O Benninga MA Beelen A van der Palen J Tamminga-Smeulders C Tijssen JG Inpatient treatment of children and adolescents with severe obesity in The Netherlands: a randomized clinical trial. JAMA Pediatr. (2014) 168(9):807–14. 10.1001/jamapediatrics.2014.521 25022831

[13] Yanovski SZ Yanovski JA. Long-term drug treatment for obesity: a systematic and clinical review. JAMA. (2014) 311(1):74–86. 10.1001/jama.2013.281361 24231879 PMC3928674

[14] Ohta M Seki Y Wong SK Wang C Huang CK Aly A Bariatric/metabolic surgery in the Asia-Pacific Region: aPMBSS 2018 survey. Obes Surg. (2019) 29(2):534–41. 10.1007/s11695-018-3539-7 30306499

[15] Aminian A Chang J Brethauer SA Kim JJ, American Society for Metabolic and Bariatric Surgery Clinical Issues Committee. ASMBS Updated position statement on bariatric surgery in class I obesity (BMI 30-35 kg/m(2)). Surg Obes Relat Dis. (2018) 14(8):1071–87. 10.1016/j.soard.2018.05.025 30061070

[16] Pratt JSA Browne A Browne NT Bruzoni M Cohen M Desai A ASMBS pediatric metabolic and bariatric surgery guidelines, 2018. Surg Obes Relat Dis. (2018) 14(7):882–901. 10.1016/j.soard.2018.03.019 30077361 PMC6097871

[17] Inge TH Courcoulas AP Jenkins TM Michalsky MP Helmrath MA Brandt ML Weight loss and health status 3 years after bariatric surgery in adolescents. N Engl J Med. (2016) 374(2):113–23. 10.1056/NEJMoa1506699 26544725 PMC4810437

[18] Inge TH Jenkins TM Xanthakos SA Dixon JB Daniels SR Zeller MH Long-term outcomes of bariatric surgery in adolescents with severe obesity (FABS-5+): a prospective follow-up analysis. Lancet Diabetes Endocrinol. (2017) 5(3):165–73. 10.1016/S2213-8587(16)30315-1 28065736 PMC8282411

[19] Vilallonga R Himpens J van de Vrande S. Long-term (7 years) follow-up of roux-en-Y gastric bypass on obese adolescent patients (&lt;18 years). Obes Facts. (2016) 9(2):91–100. 10.1159/000442758 27035348 PMC5644862

[20] White B Doyle J Colville S Nicholls D Viner RM Christie D. Systematic review of psychological and social outcomes of adolescents undergoing bariatric surgery, and predictors of success. Clin Obes. (2015) 5(6):312–24. 10.1111/cob.12119 26541244

[21] Eisenberg D Shikora SA Aarts E Aminian A Angrisani L Cohen RV 2022 American society for metabolic and bariatric surgery (ASMBS) and international federation for the surgery of obesity and metabolic disorders (IFSO): indications for metabolic and bariatric surgery. Surg Obes Relat Dis. (2022) 18(12):1345–56. 10.1016/j.soard.2022.08.013 36280539

[22] United Arab Emirates Department of Health. DOH Service Requirements for the Weight Management Program for Overweight and Obese Children. Abu Dhabi: UAE Department of Health (2018).

[23] United Arab Emirates Department of Health. DOH Standard for Obesity and Weight Diagnosis, Pharmacological and Surgical Management Interventions. Abu Dhabi: UAE Department of Health (2018).

[24] Von Elm E Altman DG Egger M Pocock SJ Gøtzsche PC Vandenbroucke JP. The strengthening the reporting of observational studies in epidemiology (STROBE) statement: guidelines for reporting observational studies. Lancet. (2007) 370(9596):1453–7. 10.1016/S0140-6736(07)61602-X 18064739

[25] McNiece KL Poffenbarger TS Turner JL Franco KD Sorof JM Portman RJ. Prevalence of hypertension and pre-hypertension among adolescents. J Pediatr. (2007) 150(6):640–444.e1. 10.1016/j.jpeds.2007.01.052 17517252

[26] Expert Panel on Integrated Guidelines for Cardiovascular H, Risk Reduction in Children and Adolescents, National Heart, Lung and Blood Institute. Expert panel on integrated guidelines for cardiovascular health and risk reduction in children and adolescents: summary report. Pediatrics. (2011) 128(Suppl 5):S213–56. 10.1542/peds.2009-2107C 22084329 PMC4536582

[27] Buysschaert M Medina JL Buysschaert B Bergman M. Definitions (and current controversies) of diabetes and prediabetes. Curr Diabetes Rev. (2016) 12(1):8–13. 10.2174/1573399811666150122150233 25612821

[28] Flegal KM Wei R Ogden CL Freedman DS Johnson CL Curtin LR. Characterizing extreme values of body mass index-for-age by using the 2000 centers for disease control and prevention growth charts. Am J Clin Nutr. (2009) 90(5):1314–20. 10.3945/ajcn.2009.28335 19776142

[29] Kelly AS Barlow SE Rao G Inge TH Hayman LL Steinberger J Severe obesity in children and adolescents: identification, associated health risks, and treatment approaches: a scientific statement from the American Heart Association. Circulation. (2013) 128(15):1689–712. 10.1161/CIR.0b013e3182a5cfb3 24016455

[30] Marginean CO Marginean C Melit LE. New insights regarding genetic aspects of childhood obesity: a minireview. Front Pediatr. (2018) 6:271. 10.3389/fped.2018.00271 30338250 PMC6180186

[31] Mocnik M Marcun Varda N. Cardiovascular risk factors in children with obesity, preventive diagnostics and possible interventions. Metabolites. (2021) 11(8):551. 10.3390/metabo11080551 34436493 PMC8398426

[32] Juonala M Magnussen CG Berenson GS Venn A Burns TL Sabin MA Childhood adiposity, adult adiposity, and cardiovascular risk factors. N Engl J Med. (2011) 365(20):1876–85. 10.1056/NEJMoa1010112 22087679

[33] Michalsky MP Inge TH Jenkins TM Xie C Courcoulas A Helmrath M Cardiovascular risk factors after adolescent bariatric surgery. Pediatrics. (2018) 141(2):e20172485. 10.1542/peds.2017-2485 29311357 PMC5810605

[34] Viner R White B Christie D. Type 2 diabetes in adolescents: a severe phenotype posing major clinical challenges and public health burden. Lancet. (2017) 389(10085):2252–60. 10.1016/S0140-6736(17)31371-5 28589895

[35] Tryggestad JB Willi SM. Complications and comorbidities of T2DM in adolescents: findings from the TODAY clinical trial. J Diabetes Complications. (2015) 29(2):307–12. 10.1016/j.jdiacomp.2014.10.009 25468310 PMC4333013

[36] Group TS Zeitler P Hirst K Pyle L Linder B Copeland K A clinical trial to maintain glycemic control in youth with type 2 diabetes. N Engl J Med. (2012) 366(24):2247–56. 10.1056/NEJMoa1109333 22540912 PMC3478667

[37] Khidir N El-Matbouly MA Sargsyan D Al-Kuwari M Bashah M Gagner M. Five-year outcomes of laparoscopic sleeve gastrectomy: a comparison between adults and adolescents. Obes Surg. (2018) 28(7):2040–5. 10.1007/s11695-018-3139-6 29430596

[38] Amin R Simakajornboon N Szczesniak R Inge T. Early improvement in obstructive sleep apnea and increase in orexin levels after bariatric surgery in adolescents and young adults. Surg Obes Relat Dis. (2017) 13(1):95–100. 10.1016/j.soard.2016.05.023 27720196 PMC5130623

[39] Kalra M Inge T Garcia V Daniels S Lawson L Curti R Obstructive sleep apnea in extremely overweight adolescents undergoing bariatric surgery. Obes Res. (2005) 13(7):1175–9. 10.1038/oby.2005.139 16076986

[40] Alqahtani A Elahmedi M Qahtani AR. Laparoscopic sleeve gastrectomy in children younger than 14 years: refuting the concerns. Ann Surg. (2016) 263(2):312–9. 10.1097/SLA.0000000000001278 26496081

[41] Ogle SB Dewberry LC Jenkins TM Inge TH Kelsey M Bruzoni M Outcomes of bariatric surgery in older versus younger adolescents. Pediatrics. (2021) 147(3):e2020024182. 10.1542/peds.2020-024182 33526606 PMC7919111

[42] Jaklevic MC. The push for earlier bariatric surgery for adolescents with severe obesity. JAMA. (2021) 325(22):2241–2. 10.1001/jama.2021.7912 34014255

[43] Vuong L Chang SH Wan F Wu N Eagon JC Eckhouse SR National trends and outcomes in adolescents undergoing bariatric surgery. J Am Coll Surg. (2022) 235(2):186–94. 10.1097/XCS.0000000000000234 35839393

[44] Mocanu V Lai K Dang JT Switzer NJ Birch DW Ball GDC Evaluation of the trends, characteristics, and outcomes in North American youth undergoing elective bariatric surgery. Obes Surg. (2021) 31(5):2180–7. 10.1007/s11695-021-05248-6 33548012

[45] Poliakin L Roberts A Thompson KJ Raheem E McKillop IH Nimeri A. Outcomes of adolescents compared with young adults after bariatric surgery: an analysis of 227,671 patients using the MBSAQIP data registry. Surg Obes Relat Dis. (2020) 16(10):1463–73. 10.1016/j.soard.2020.05.028 32709580

[46] Janson A Jarvholm K Gronowitz E Sjogren L Klaesson S Engstrom M A randomized controlled trial comparing intensive non-surgical treatment with bariatric surgery in adolescents aged 13-16 years (AMOS2): rationale, study design, and patient recruitment. Contemp Clin Trials Commun. (2020) 19:100592. 10.1016/j.conctc.2020.100592 32637723 PMC7330152

[47] Younes S Ibrahim A Al-Jurf R Zayed H. Genetic polymorphisms associated with obesity in the Arab world: a systematic review. Int J Obes (Lond). (2021) 45(9):1899–913. 10.1038/s41366-021-00867-6 34131278 PMC8380539

[48] Bordoni L Marchegiani F Piangerelli M Napolioni V Gabbianelli R. Obesity-related genetic polymorphisms and adiposity indices in a young Italian population. IUBMB Life. (2017) 69(2):98–105. 10.1002/iub.1596 28090739

[49] Loktionov A. Common gene polymorphisms and nutrition: emerging links with pathogenesis of multifactorial chronic diseases (review). J Nutr Biochem. (2003) 14(8):426–51. 10.1016/s0955-2863(03)00032-9 12948874

[50] Badran M Laher I. Obesity in Arabic-speaking countries. J Obes. (2011) 2011:686430. 10.1155/2011/686430 22175002 PMC3228340

[51] Alqout O Reynolds F. Experiences of obesity among Saudi Arabian women contemplating bariatric surgery: an interpretative phenomenological analysis. J Health Psychol. (2014) 19(5):664–77. 10.1177/1359105313476977 23479306

[52] Steinberger AE Youngwirth LM Kim SE Duke NN Skinner A Gordee A Adolescent bariatric surgery: racial disparities in 30-day outcomes using the MBSAQIP from 2015 to 2018. Obes Surg. (2021) 31(8):3776–85. 10.1007/s11695-021-05500-z 34043179 PMC8895205

[53] Abusnana S Abdi S Tagure B Elbagir M Maleckas A. Bariatric surgery outcomes: a single-center study in the United Arab Emirates. Diabetes Metab Syndr Obes. (2015) 8:461–71. 10.2147/DMSO.S87861 26425103 PMC4583119

[54] Al Mansoori A Shakoor H Ali HI Feehan J Al Dhaheri AS Cheikh Ismail L The effects of bariatric surgery on vitamin B status and mental health. Nutrients. (2021) 13(4):1383. 10.3390/nu13041383 33923999 PMC8073305

[55] Kelly AS Auerbach P Barrientos-Perez M Gies I Hale PM Marcus C A randomized, controlled trial of liraglutide for adolescents with obesity. N Engl J Med. (2020) 382(22):2117–28. 10.1056/NEJMoa1916038 32233338

